# Investigating the Impact of the Parkinson’s-Associated GBA1 E326K Mutation on β-Glucocerebrosidase Dimerization and Interactome Dynamics Through an In Silico Approach

**DOI:** 10.3390/ijms252111443

**Published:** 2024-10-24

**Authors:** Davide Pietrafesa, Alessia Casamassa, Barbara Benassi, Massimo Santoro, Massimo Marano, Claudia Consales, Jessica Rosati, Caterina Arcangeli

**Affiliations:** 1 Department of Biology, University of Rome Tor Vergata, Via della Ricerca Scientifica 1, 00133 Rome, Italy; davide.pietrafesa@uniroma2.it; 2 PhD Program in Materials for Health, Environment and Energy, University of Rome Tor Vergata, Via della Ricerca Scientifica 1, 00133 Rome, Italy; 3 Cellular Reprogramming Unit, Fondazione IRCCS Casa Sollievo della Sofferenza, Viale dei Cappuccini 1, 71013 San Giovanni Rotondo, Italy; a.casamassa@css-mendel.it (A.C.); or jessicadiana.rosati@unicamillus.org (J.R.); 4 Division of Biotechnologies, Department for Sustainability, Italian National Agency for New Technologies, Energy and Sustainable Economic Development (ENEA), Via Anguillarese 301, 00123 Rome, Italy; barbara.benassi@enea.it (B.B.); massimo.santoro@enea.it (M.S.); claudia.consales@enea.it (C.C.); 5 Unit of Neurology, Neurophysiology, Neurobiology and Psychiatry, Department of Medicine, University Campus Biomedico, Via Alvaro del Portillo 200, 00128 Rome, Italy; m.marano@policlinicocampus.it; 6 Fondazione Policlinico Universitario Campus Biomedico, Via Alvaro del Portillo 200, 00128 Rome, Italy; 7 Departmental Faculty of Medicine, UniCamillus, Saint Camillus International University of Health Sciences, Via di Sant’Alessandro 8, 00131 Rome, Italy

**Keywords:** molecular dynamics simulation, Gaucher disease, GCase dimer structure, pH effect, Sap C, α-Syn, LIMP-2

## Abstract

Heterozygous mutations or genetic variants in the *GBA1* gene, which encodes for the β-glucocerebrosidase (GCase), a lysosomal hydrolase enzyme, may increase the risk of Parkinson’s disease (PD) onset. The heterozygous E326K form is one of the most common genetic risk factors for PD worldwide, but, to date, the underlying molecular mechanisms remain unclear. Here, we investigate the effect of the E326K on the structure, stability, dimerization process, and interaction mode with some proteins of the interactome of GCase using multiple molecular dynamics (MD) simulations at pH 5.5 and pH 7.0 to mimic the lysosomal and endoplasmic reticulum environments, respectively. The analysis of the MD trajectories highlights that the E326K mutation did not significantly alter the structural conformation of the catalytic dyad but significantly makes the structure of the dimeric complexes unstable, especially at lysosomal pH, potentially impacting the organization of the quaternary structure. Furthermore, the E326K mutation significantly impacts protein interactions by altering the binding mode with the activator Saposin C (SapC), reducing the binding affinity with the inhibitor α-Synuclein (α-Syn), and increasing the affinity for the Lysosomal integral membrane protein-2 (LIMP-2) transporter.

## 1. Introduction

**Figure 1:**
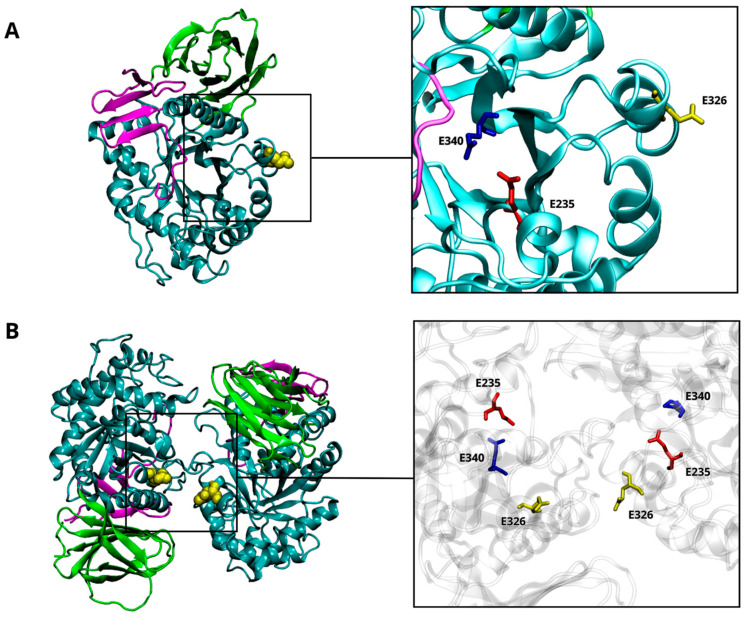
β-glucocerebrosidase (GCase) structure. The 3D structure of the GCase (PDB-ID: 3GXI) showing domain I (magenta), domain II (green), and domain III (cyan). The catalytic dyad is highlighted by a squared box showing the E235 (red) and the E340 (blue) residues. The site of mutation (E326) is also highlighted in yellow (**A**). The 3D structure of the wild-type CGase dimer. The interaction’s interface between the two monomers is highlighted by a squared box that shows the E235 (red), the E340 (blue), and the E326 (yellow) residues (**B**).

The correlation between the risk of developing Parkinson’s disease (PD) and mutations in the *GBA1* gene has been known since the 1990s, when PD cases were first observed in Ashkenazi Jewish (AJ) patients with Gaucher’s disease (GD) and in their family members [1,2]. GD is the most common autosomal recessive lysosomal storage disorder, in which the glycolipid substrate accumulates in cells. GD is caused by the defective activity of the *GBA1*-encoded β-glucocerebrosidase (GCase), a lysosomal enzyme. GCase is a glycoprotein synthesized in the endoplasmic reticulum (ER), where it interacts with the lysosomal integral membrane protein 2 (LIMP-2) through hydrophobic interfaces, facilitating its transport to the lysosome. Here, GCase catalyzes the hydrolysis of glucosylceramide (GlcCer) into ceramide and glucose with the help of the activator protein Saposin C (SapC). SapC, a glycoprotein derived by proteolytic cleavage of Prosaposin [3], facilitates the interaction of GCase with its natural substrates and induces a conformational change that directly stimulates the enzyme’s activity [4,5]. The structure of the glycoprotein GCase is characterized by three discontinuous domains (Figure 1A). Domain I is an antiparallel β-sheet flanked by a loop, while domain II forms an eight-stranded β-barrel organized in a manner reminiscent of the folding assumed by immunoglobulins [6]. Domain II plays a regulatory and structural function, even though most of the mutations associated with GD are situated in domain III, which is composed of a (β/α)-8-barrel of triose phosphate isomerase (TIM). This latter domain contains the catalytic dyad consisting of two glutamic residues: E340, acting as the nucleophile, and E235, functioning as the acid/base catalyst. At the entrance of the active catalytic site, there are five exposed loops [7,8,9]. The rearrangements of these loops, pH conditions, and specific residue interactions collectively influence the accessibility of the catalytic site, determining the active and inactive states of the GCase [10].

More than 490 *GBA1* mutations are known to be associated with GD, but only a few of them (20–30%) have been so far associated with PD [11]. Mutations linked to a pathological outcome are spread throughout the GCase structure. Many are located far from the active site but still ensure a certain degree of enzymatic activity. Other mutations are situated close to the regions responsible for interaction with either the SapC activator or the LIMP-2 transporter. Nevertheless, a clear correlation between the location of mutation and the severity of the diseases has not been established yet. E326K, E388K, and T369M *GBA1* mutations do not cause GD but have been specifically associated with an increased risk (i.e., “risk” variants) of developing aggressive α-synucleinopathies, such as PD and dementia with Lewy bodies. Specifically, E326K is situated far from the catalytic site (Figure 1A) and predisposes to PD onset in both homozygous and heterozygous forms [12,13,14], thus increasing the risk by up to 10 times [11]. Some heterozygous GCase mutations promote PD pathogenesis through a gain-of-function mechanism that may include ER stress [15]. This implies a multifactorial GBA/PD-based molecular mechanism that does not depend solely on the deficient lysosomal activity. Recent in vitro findings demonstrated that the E326K mutation in human fibroblasts does not significantly reduce enzymatic activity, whereas it stimulates the aggregation of insoluble α-Synuclein (α-Syn) fibrils and lipid droplet formation [16], a set of structural modifications typically characterizing human PD neurons [17,18,19]. Moreover, the accumulation of a high amount of GlcCer may contribute to α-Syn aggregation, leading to decreased GCase activity [11]. Despite in vitro experimental evidence supporting the occurrence of a specific α-Syn-GCase complex, no data have been provided in terms of identification of the molecular mechanism(s) underlying such interaction. A direct interaction of the α-Syn’s C-terminus has been postulated to occur with specific residues of the GCase in its monomeric form to promote lysosomal degradation of α-Syn or inhibit its excessive accumulation [20,21]. The wild-type GCase displays a more stable and functional structure as a dimer, where the active sites are partially buried [22]. The GCase dimer has a butterfly-shaped structure (Figure 1B), and the dimer interface provides an allosteric binding pocket [22,23]. It was suggested that dimerization could be an important process for GCase activation [23]. Considering the translocation of the GCase from the ER to the lysosome, other studies highlighted the presence of dimers at neutral and acidic pHs both in vitro [23] and in vivo [24]. The equilibrium is mainly altered by interaction with SapC, shifting toward the monomeric form [24]. It is not clear whether SapC acts by dissociating the GCase dimer or by binding the free monomer, preventing self-association.

Despite efforts to find relationships between mutations, protein structure, GD manifestation, and enzymatic activity in the literature, our understanding of how specific mutations alter GCase’s tertiary and quaternary structure and its interaction with other proteins and influence the risk of developing PD remains incomplete. To such an extent, a computational approach based on molecular dynamics (MD) simulations can help in understanding the differences in the structural and functional behavior of a protein due to mutations. Here, to gain insight into the effect of the E326K mutation on the tertiary and quaternary structure of GCase, we employed several classical MD simulations. They act as a computational microscope able to capture the behavior of biomolecules and dynamically map their interactions in full atomic detail [25]. Heterodimeric, wild-type, and mutated homodimeric complexes of GCase were modeled and simulated at both pH 5.5 and 7.0, mimicking the lysosomal and ER environments, respectively. The potential effects of E326K mutation on the active site of the GCase, the stability of its dimerization, and its interaction dynamics with LIMP-2, SapC, and α-Syn were investigated and evaluated through interaction energy characterization. Our computational approach revealed that the E326K mutation does not affect the structural integrity of the catalytic site but reduces binding affinity with the inhibitor α-Syn and destabilizes dimeric complexes, particularly under lysosomal pH conditions.

## 2. Results

Here, we report a comprehensive analysis of the structure stability and interactome dynamics of wild-type and mutated GCase dimers by exploiting classical MD simulations, which provide unique and useful insights into the structural stability and dynamics of several biological systems [25,26,27,28].

### 2.1. The E326K Mutation Does Not Affect the Catalytic Dyad’s Structure

The site of mutation is sufficiently far from the catalytic dyad (Figure 1), and this might have no direct effect on it. To verify this hypothesis, we monitored throughout the simulations at pH 5.5 (i) the distances between the mutation site (residue 326) and the two catalytic residues (E340 and E235), as well as (ii) the distance between the two residues that form the catalytic dyad; their averaged values are reported in Table 1. The average distances between site 326 and residue E235 are comparable in wild-type and mutant monomers; in the mutated one, a higher distance between K326 and E340 is observed compared to the wild-type monomer. This may be attributed to the mutation, which affects the accessibility of the catalytic site, potentially increasing its available surface area. Nevertheless, the mutation does not significantly affect the average distance between the two catalytic residues (E235 and E340). The preservation of this distance can be considered a reliable indicator of the structural integrity and retention of catalytic competence in GCase.

**Figure 2:**
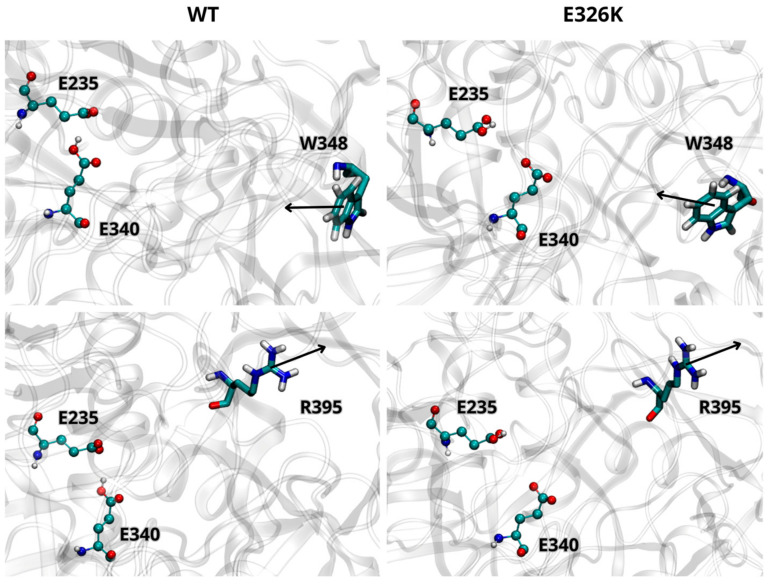
Orientation of W348 and R395 residues in wild-type (WT) and mutated GCase at pH 5.5. Arrows indicate the direction of the side chain. The images are representative snapshots from the simulations. Color codes for the selected residues: carbon, cyan; hydrogen, white; nitrogen, blue; oxygen, red.

**Figure 3:**
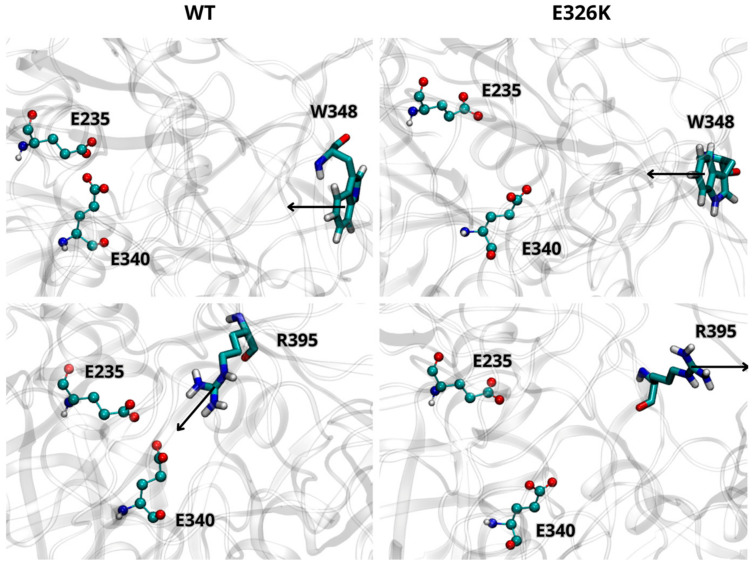
Orientation of residues W348 and R395 in WT and mutated GCase at pH 7.0. Arrows indicate the direction of the side chain. The images are representative snapshots from the simulations. Color codes for the selected residues: carbon, cyan; hydrogen, white; nitrogen, blue; oxygen, red.

We further analyzed the effect of the E326K mutation on the catalytic dyad by monitoring the side chain orientations of residues W348 and R395 relative to E340 and E235. These residues are known to adopt different orientations depending on the activity state of GCase [10]. In the inactive state at a neutral pH, W348 blocks the catalytic dyad by pointing toward the active site, while R395 faces E340. At an acidic lysosomal pH, W348 shifts toward the active site, and R395 points outward. As shown in Figure 2, the side chain orientations of W348 and R395, in both wild-type and mutated monomers (at acidic pH), are in agreement with data reported in the literature [29], indicating that the E326K mutation does not impair GCase functionality in the lysosome. Interestingly, in the inactive state at pH 7.0 (Figure 3), R395 behaves as expected in the wild-type monomer, while in the mutated one, its orientation deviates. This suggests that catalytic E340 residue may not be properly blocked, potentially compromising the inactive form of GCase at a neutral pH.

We monitored specific hydrogen bonds that play crucial roles in GCase activation, focusing on the Y313-E235 bond at pH 7.0 and the Y313-E340 bond at an acidic pH (Table 2, Figure S1). At a neutral pH, the Y313-E235 bond acts as a gate, keeping the active site closed [6,9]. As expected, such a bond is present in the wild-type GCase with over 47% persistence, but it disappears in the mutated protein at pH 7.0. Upon GlcCer substrate binding at lysosomal pH, Y313 shifts to bind with E340 in the active site, suggesting an induced-fit mechanism for GCase near the active site [6,9]. In simulations at an acidic pH without the substrate, the Y313-E340 bond is absent in the wild-type protein, as anticipated, but unexpectedly, it is present in the mutated GCase, persisting for over 47% of the simulation (Table 2, Figure S1).

At a neutral pH, the E326 residue forms three stable hydrogen bonds with a persistence of over 70% during the simulation. The mutation from E to K triggers the loss of two of them (Table 3, Figure S2). Despite this change, the total number of hydrogen bonds is higher in the mutant than in the wild-type form at both pH 7.0 and pH 5.5 (Table 4).

### 2.2. GCase Dimerization Is Disfavoured by the E326K Mutation

It has been proposed that dimerization may play a crucial role in the functioning and activation of GCase, with the dimeric form proving to be more stable and functional than the monomeric one [22,23,24]. To assess the impact of the E326K mutation on dimer stability, we monitored the interchain hydrogen bond pattern at an acidic (Table 5) and neutral pH (Table 6). While residue 326 is located at the dimer interface (Figure 1), it does not directly contribute to the intrachain hydrogen bond network in any of the simulations analyzed. At an acidic pH (Table 5), notably, in the homodimer wild-type, residue R395 forms a hydrogen bond with E349, part of loop 1 at the entrance of monomer B’s binding site. This bond is absent in the mutated dimers. As reported in Table 5, the mutation significantly reduces the number of hydrogen bonds, indicating destabilization of the mutated homodimer in the lysosomal environment. At pH 7.0, however, the mutated homodimer shows an increase in hydrogen bond interactions compared to the wild-type dimer (Table 6).

The instability of the E326K homodimer at pH 5.5 is further supported by PCA-based conformational analysis (Figure S4), which shows that, while the wild-type homodimer predominantly occupies a single stable conformational state with minimal variability, the E326K homodimer exhibits a broader spread along PC1. This suggests increased flexibility and a potentially less stable state.

The strength of interaction between monomers in the complexes was estimated by calculating the binding free energy (ΔG) for each system, with the results listed in Table 7. At pH 5.5, the wild-type homodimer exhibits significantly lower binding energy values (higher affinity) compared to those at pH 7.0, in line with the literature [23,24], which suggests stronger monomer interactions at an acidic pH to support the protein’s hydrolytic function. The E326K mutation has a destabilizing effect on the mutated homodimer, particularly at pH 5.5, where the binding energy is substantially less favorable (−14.55 ± 0.43 kcal/mol) than that of the wild-type homodimer (−18.89 ± 0.43 kcal/mol) and the heterodimer (−16.88 ± 0.22 kcal/mol). At pH 7.0, the wild-type homodimer again shows the highest binding affinity (−15.70 ± 0.16 kcal/mol), followed by the WT-E326K heterodimer (−14.99 ± 0.24 kcal/mol) and the mutated E326K-E326K homodimer (−14.72 ± 0.25 kcal/mol). This may have a negative effect on the trafficking of dimeric GCase in neutral pH vesicles [23].

### 2.3. The Effects of E326K on GCase Interactome

**Figure 4:**
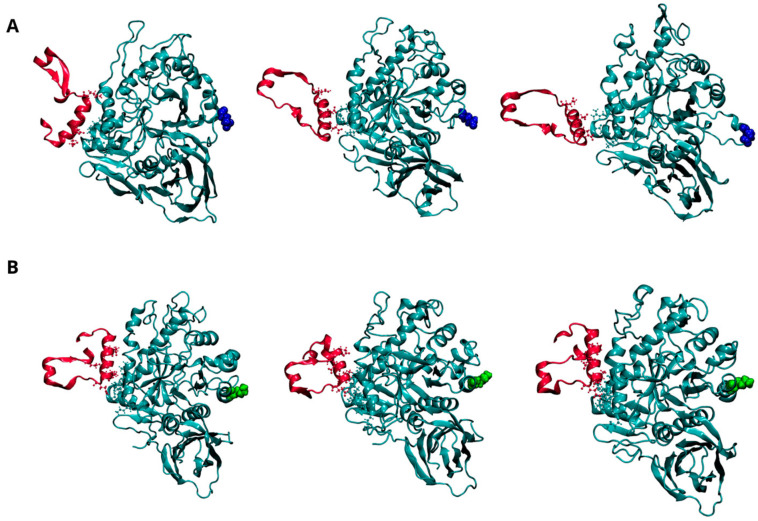
Dynamics of interaction between GCase and Lysosomal integral membrane protein-2 (LIMP-2) at pH 7.0. Representative snapshots from the concatenated molecular dynamics (MD) trajectories of the WT-LIMP-2 complex (**A**) and the E326K-LIMP-2 complex (**B**). GCase is shown in cyan, and LIMP-2 is in red, both depicted in a New Cartoon representation. Key residues involved in the interaction between the two proteins are displayed in the CPK model. The mutation site, residue 326, is highlighted in blue for the wild-type GCase and green for the mutated GCase, represented in the VDW model. For clarity, the water molecules are not shown, and only the LIMP-2 region interacting with GCase is shown here; the full complex can be viewed in Figure S5 of the Supplementary Materials.

**Figure 5:**
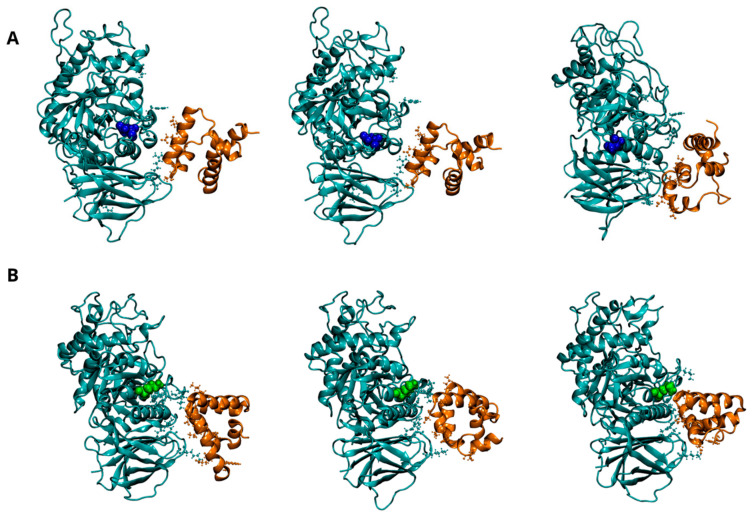
Dynamics of interaction between GCase and Saposin C (SapC) at pH 5.5. Representative snapshots from the concatenated MD trajectories of the WT-SapC complex (**A**) and the E326K-SapC complex (**B**). GCase is shown in cyan and SapC-2 in orange, both depicted in a New Cartoon representation. Key residues involved in the interaction between the two proteins are displayed in the CPK model. The mutation site, residue 326, is highlighted in blue for the wild-type GCase and green for the mutated GCase, represented in the VDW model. For clarity, the water molecules are not shown.

**Figure 6:**
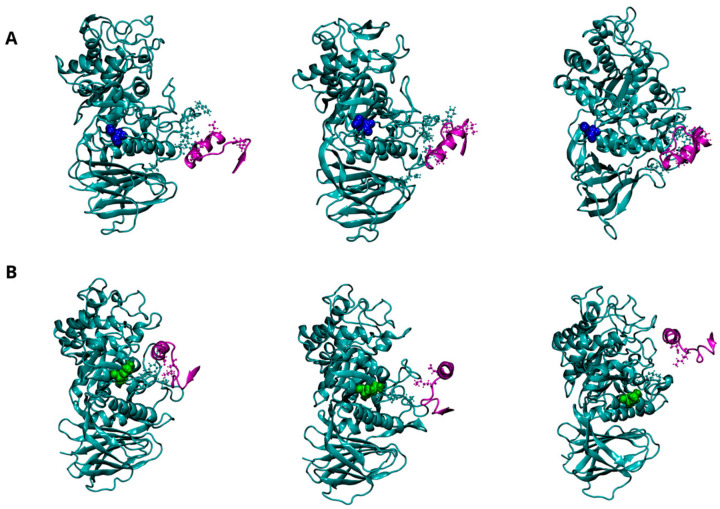
Dynamics of interaction between GCase and α-Synuclein (α-Syn) at pH 5.5. Representative snapshots from the concatenated MD trajectories of the WT-α-Syn complex (**A**) and the E326K-α-Syn complex (**B**). GCase is shown in cyan, and α-Syn is in purple, both depicted in a New Cartoon representation. Key residues involved in the interaction between the two proteins are displayed in the CPK model. The mutation site, residue 326, is highlighted in blue for the wild-type GCase and green for the mutated GCase, represented in the VDW model. For clarity, the water molecules are not shown.

The potential impact of the E326K mutation on the dynamics of interaction between GCase and its interactor proteins (LIMP-2, SapC, and α-Syn) was investigated. Evidence from both in vivo and in vitro studies suggests that GCase exists as a dimer in both the ER and lysosome [24]. To date, it is unclear whether these interacting proteins bind GCase in its monomeric or multimeric form. Experimental findings indicate that GCase binds to LIMP-2 and SapC in a 1:1 stoichiometry [24,30] and that SapC may disrupt the multimer by binding near the active site [24]. Additionally, therapeutic chaperones [22,23] act as dimer stabilizers, which seems to enhance enzymatic activity, even in the presence of mutations. A plausible hypothesis, therefore, is that interactome proteins might bind dimeric GCase through a mechanism where the ligand competes with one of the monomers. In our docking simulations, we used the monomer extracted from the dimeric structure, followed by molecular dynamics, allowing the proteins to further refine their interaction interface. A visual inspection of the molecular configurations sampled by the complexes is shown in Figure 4, Figure 5 and Figure 6 at different points of the concatenated trajectories.

Figure 4A (and Figure S5A) shows the dynamic behavior of the interaction interface between LIMP-2 and the wild-type GCase monomer at pH 7.0. As reported in the literature, the interaction involves residues from 152 to 175 of LIMP-2 and several segments of GCase (86–96, 99–110, and 150–168) [30], all located on the opposite side from the mutation site at position 326. As shown in Figure 4B (and Figure S5B), the E326K monomer interacts with a greater number of residues, indicating a stronger and more stable interaction with LIMP-2.

This observation is reinforced by the analysis of intermolecular interactions (hydrogen bonds and salt bridges) shown in Table 8 and Figure S6. The data show an increased number of hydrogen bonds between the mutant and LIMP-2, although a salt bridge between R163 of GCase and E149 of LIMP-2 is lost.

At the lysosomal level, GCase interacts with the activator SapC to catalyze the hydrolysis of GlcCer [4,5]. Although the exact interaction interface between GCase and SapC is not fully defined, experimental and computational studies suggest that residues 441–445, 463–466, and 487 of GCase are critical for binding SapC [29]. SapC has two key binding domains: domain 1 (residues 6–34) and domain 2 (residues 41–70), with domain 2 known to bind GCase with significantly higher affinity [31,32]. Figure 5A shows the potential interaction interface between SapC and wild-type GCase, showing that most interactions occur with domain 2 of SapC (residues 44 to 70). The E326K changes the interaction pattern, leading to increased interactions with domain 1 (residues 6–27) of SapC. The hydrogen bond patterns between chains, as detailed in Table 9 and Figure S7, support this finding and suggest that the mutation may alter the mode of interaction with SapC.

Three putative regions of GCase (223, 273–328, and 344–349) are thought to interact with the C-terminus of α-Syn (residues 118 to 137). Although a direct interaction with GCase has been proposed to promote lysosomal degradation of α-Syn or inhibit its excessive accumulation, it is still unclear which of these regions plays a predominant role in the interaction or the specific mechanism through which GCase interacts with α-Syn. Figure 6A illustrates the potential interaction interface between wild-type GCase and α-Syn, indicating that the 344–349 region is directly involved in the interaction. Interestingly, these interactions become extremely weak in the mutant, suggesting that the mutation at position 326 severely affects GCase’s ability to stably bind α-Syn (Figure 6B). The significant loss of affinity for α-Syn can be clearly attributed to the reduction of several hydrogen bonds and two salt bridges, which are the driving forces behind intermolecular interactions, as shown in Table 10 and Figure S8.

To quantify the strength of the interaction between GCase and LIMP-2, SapC, and α-Syn, the binding free energy (ΔG) for each system was predicted using the PRODIGY server. The results, presented in Table 11, demonstrate that the GCase mutant significantly decreases its binding affinity for α-Syn (−7.47 ± 0.50 kcal/mol) compared to the wild-type form (−9.21 ± 0.21 kcal/mol), while it appears to have lower binding energy values, therefore higher affinity, with LIMP-2. In contrast, the ΔG values for the interaction with SapC are comparable.

## 3. Discussion

*GBA1* mutations are the leading genetic risk factor for the development of PD [11,33]. Here, we focused on the E326K mutation, which is recognized for increasing the susceptibility to PD in affected individuals [33]. However, to date, the impact of such mutation on the protein structure, as well as on physiological functions, remains unclear. MD simulation is acknowledged for its ability to evaluate the stability, flexibility, and dynamics of molecular systems at both temporal and spatial resolutions, making it a powerful investigative tool [25,26,27,28]. In this study, we employed classical MD simulations to identify the pathological mechanism, at the structural level, of the E326K mutation of the *GBA1* gene. We accounted for the heterozygous condition by simulating three distinct GCase dimers: a mutant heterodimer, as well as wild-type and mutant homodimers. To the best of our knowledge, we present the first computational study addressing i) the characterization of the structural, dynamical, and physicochemical features of these dimers at two different pH levels—representing the lysosomal (acid pH) and ER (neutral pH) environments—as well as ii) the interaction models between GCase and its interactome. Our findings demonstrate that the proposed computational approach serves as a valuable tool for assessing the structural features of the GCase in physiological aqueous solutions, along with their conformational rearrangements at an atomic resolution.

The structural analysis of the GCase monomers shows that the E326K mutation does not significantly affect the catalytic dyad (E235-E340), consistent with experimental in vitro findings by Smith et al. (2022) [16], which showed that the E326K mutation in human fibroblasts does not substantially reduce enzymatic activity. But, significantly, the E326K mutation may compromise the inactive state of GCase at a neutral pH. It has been observed that E326K does not affect the orientation of residues W348 and R395, which plays a crucial role at specific pH levels during GCase function. Notably, the orientation of these two residues at an acidic pH is not influenced. On the contrary, the side chain orientation of R395 deviates from the observed orientation [8,9,29] under neutral pH conditions. Based on this result, it could be hypothesized that the transition of GCase from an inactive to an active form and vice versa, regulated by various cellular factors, does not occur, potentially because the enzyme is in continuous activity. The lack of the Y313-E235 hydrogen bond, which acts as a gate, in the mutated GCase does not allow the inactive form of the GCase to be maintained at a neutral pH.

Given the uncertainty about whether the enzyme exists as a monomer or dimer in the cellular context [23], we focused attention on the formation of dimers, observing the mutation’s influence on the spatial arrangement of the systems. Overall, the results suggest a potential mutation-induced destabilizing effect on dimeric structure. At the structural level, the mutated homodimer appears disadvantaged compared with the wild-type one. This effect becomes more pronounced with a decreasing pH from neutral to acidic, as indicated by hydrogen bond analysis. This result suggests that the E326K mutation has a major impact under lysosomal conditions, i.e., when GCase both explicates its enzymatic activity and interacts with other proteins.

Since GCase interacts with various proteins during its journey from the ER to the lysosome and within the lysosomal environment, in this study, we simulated the dynamics of interaction between GCase and its interactome to reveal if the mutation causes gain-of-function and loss-of-function effects. Taken together, our results suggest that the mutation exerts a dual gain-of-function and loss-of-function mechanism. The LIMP-2 transporter may retain the mutated form of GCase, which exhibits greater affinity than the wild type (gain-of-function), potentially inducing endoplasmic reticulum stress. Simultaneously, a loss-of-function mechanism is triggered due to the significantly reduced interaction between mutant GCase and α-Syn, leading to α-Syn accumulation and the disruption of its physiological competition with SapC. This loss of direct GCase-α-Syn interaction may explain the aggregation of insoluble α-Syn fibrils caused by the E326K mutation, as observed in in vitro experiments on human fibroblasts [16]. Additionally, the interaction with its activator, SapC, also seems to be affected by the presence of the mutation. We would like to stress that in this study, we focused on simulating interactions between interactome proteins and the monomeric form of GCase. However, it is possible that interactome proteins interact with dimeric GCase via a competitive mechanism, wherein proteins like α-Syn, SapC, or LIMP-2 may compete with one of the monomers. This competition suggests that interactome proteins might disrupt the dimer, effectively replacing one GCase monomer, as has been reported in the literature for SapC [24]. Consequently, the complexes we simulated represent a plausible state following the dissociation of one monomer. Further research would be valuable to investigate whether interactome proteins stabilize this dissociation or bind directly in a way that destabilizes the dimer, potentially shedding light on a dynamic equilibrium between monomeric and dimeric forms in cellular environments.

If experimentally validated, these findings provide the groundwork to explore molecular mechanisms underlying the correlation between the E326 mutation and PD onset.

## 4. Materials and Methods

### 4.1. Structures’ Preparation

The 3D structure of the wild-type dimeric GCase (Uniprot ID P04062) was obtained by selecting the A and B chains of the 3GXI crystal (X-ray resolution: 1.34 Å) [6]. To model the heterozygous condition, two additional dimeric complexes were prepared: the mutant homodimer and the heterodimer, in which the E326K mutation was introduced, through the CHARMM-GUI platform (https://www.charmm-gui.org/, accessed on 11 January 2024) [34,35,36]. The mutation was introduced in both the chains for the homodimer and in only the A chain for the heterodimer. The protonation states of titratable amino acids at pH 5.5 and pH 7.0 were predicted and determined with PROPKA 3.1 by means of the Play Molecule tool (https://open.playmolecule.org/, accessed on 11 January 2024) [37] and listed in Supplementary Table S9. The wild-type and mutated GCase monomers at pH 5.5 were docked against the 3D structure of SapC (Uniprot ID P07602) in the open conformation (PDB id: 2QYP, chain A) [38] and of α-Syn (Uniprot ID P37840) fused to the maltose binding protein (PDB id: 3Q27) [39] to create complexes that simulate lysosomal interactions. At neutral pH, the wild-type and mutated GCase monomers were docked with the 3D structure of LIMP-2 (Uniprot ID Q14108, PDB id: 4F7B) [40] to model their interaction in the endoplasmic reticulum. Docking calculations were performed using the HADDOCK version 2.4 protein–protein docking algorithm [41], incorporating known active residues as data restraints to guide simulations. Table 12 summarizes the prepared systems: a total of twelve (12) complexes were set up as starting systems for the classical MD simulations.

### 4.2. MD Simulations

All the MD simulations were performed by using Gromacs version 2019 [42,43] with the GROMOS 53a6 united-atoms force field [44]. The systems were solvated by using TIP3P model [45], neutralized with NaCl (0.15 M), and energy-minimized by using 5000 steps of the steepest descent method [46]. Periodic boundary conditions (PBC) were applied to avoid edge effects and to better describe the full hydration conditions. Systems were equilibrated for the first 200 ps under the NVT ensemble at 100 K, followed by further 200 ps under NPT ensemble at 310 K, following the protocol shown in Supplementary Table S10. The time step was set to 2 fs. The V-rescale algorithm [47] with a time constant of 0.1 was used to keep the temperature constant. The average pressure was kept constant to 1 bar by using the Parrinello–Rahman barostat [48]. The LINCS algorithm [49] was used to constrain all the bonds throughout the equilibration process. Unrestrained replica MD simulations with a time step of 2 fs were carried out for each system (Table 12). The replicates are simulations of identical structures with identical parameters where only the initial velocities are created randomly according to a Maxwell–Boltzmann distribution. Long-range electrostatic interactions were computed using the Particle Mesh Ewald (PME) method [50]. A residue-based cutoff of 1 nm was used for the short-range electrostatic and van der Waals interactions. All MD simulations were performed using High Parallel Computing (HPC) provided by the computational infrastructure ENEAGRID/CRESCO (Computational RESearch Center of COmplex systems) [51] site in the ENEA Research Centre of Portici, Italy.

### 4.3. Analysis of the Trajectories

The first 400 ns of each dimer’s replica were excluded as equilibration phase. The remaining frames were concatenated to form a continuous trajectory for each system. Similarly, for the GCase-protein interactor complexes, the first 50 ns of each trajectory were discarded before concatenation. The concatenated trajectories were analyzed using modules included in the GROMACS suite to extract various structural, dynamic, and energetic features. The *mindist* module was employed to monitor the minimum distance between the center of mass of the A and B chains and between specific residues. Cluster analysis was conducted using the *cluster* module and by using the gromos method [52]. A matrix of atom positional RMSD was calculated for the Cα atoms of the proteins. Structural similarity was assessed using a positional RMSD cutoff value determined from the RMSD distribution curve for each system. The structures identified by the most populated clusters were used as representative snapshots for the visualization analysis and for ΔG_binding_ analysis using the PRODIGY (PROtein binDIng enerGY prediction) server (https://rascar.science.uu.nl/prodigy/, accessed on 19 June 2024) [53], which provides a quantitative estimate of the binding affinity between the A and B chains of the complexes by analyzing intermolecular contacts and desolvation effects. It specifically evaluates the number of contacts between the binding partners within a defined distance threshold, factoring in temperature as a correction. The free energy (ΔG) is then calculated based on the number and type of intermolecular contacts (ICs) at the interface, classifying them into polar, apolar, and charged interactions. This method relies on statistical models derived from experimental data, offering a reliable prediction of binding affinities while acknowledging the inherent approximations of empirical approaches [53]. Hydrogen bonds and the salt bridges were identified with the *H-bonds* and *Salt Bridge* VMD version 1.9.3 plugins, respectively [54]. A hydrogen bond was assumed to exist if the donor to acceptor distance was shorter than 0.35 nm and the hydrogen donor–acceptor angle was lower than 30°. The hydrogen bond persistence throughout a simulation was calculated for evaluating the duration, or “lifetime”, of specific hydrogen bonds. In our analysis, we specifically considered hydrogen bonds that persisted beyond a defined percentage of the simulation time, as specified in the legend of tables. A salt bridge was assumed to exist if the distance between the side-chain oxygen (O) of Asp or Glu and the side-chain nitrogen (N) of Lys, Arg, or His was less than 0.50 nm. Principal component analysis (PCA) of the covariance matrix of the positional fluctuations of the Cα atoms [55] was performed by using the *covar* module. The covariance matrix was built from each replica trajectory of the dimers, from which the overall rotational and translational motions were removed. The *anaeig* module was used to calculate the 2D projections with respect to the first two eigenvectors. All graphical representations were generated using VMD version 1.9.3 [54].

## Figures and Tables

**Table 1:** Time-averaged distances (nm) between specific residues. Notes: data are presented as mean ± standard deviation. For comparison, the average values obtained when the enzyme is in its inactive state at pH 7.0 are provided in Table S1 of the Supplementary Materials.

Simulation at pH 5.5	Wild-Type	E326K
E/K326-E235	2.04 ± 0.08	2.13 ± 0.08
E/K326-E340	2.03 ± 0.06	2.38 ± 0.08
E235-E340	0.73 ± 0.06	0.77 ± 0.04

**Table 2:** Hydrogen bonds between specific residues of the active site.

Pair Residues, pH	Wild-Type	E326K
Y313-E235, pH 7.0	67.70 ^a^	--
Y313-E340, pH 5.5	--	47.80

^a^ The number indicates the percentage (%) of persistence of hydrogen bonds throughout the concatenated simulation. The value was calculated as the average of the persistence percentages of individual replicas that are reported in Table S2. Only the hydrogen bonds with a persistence of over 45% were considered. These hydrogen bonds are depicted in Figure S1.

**Table 3:** Hydrogen bonds involving the mutation site, residue 326 at neutral pH.

pH 7.0	Wild-Type	E326K
Residue/Residue	E326	K326
H328	70.82 ^a^	--
R329	82.43	--
L330	76.72	80.78

^a^ The number indicates the percentage (%) of persistence of hydrogen bonds throughout the concatenated simulation. The value was calculated as the average of the persistence percentages of individual replicas that are reported in Table S3. Only the hydrogen bonds with a persistence of over 70% were considered. These hydrogen bonds are depicted in Figure S2.

**Table 4:** Time-averaged number of intrachain hydrogen bonds.

pH	Wild-Type	E326K
pH 5.5	37 ± 0.17 ^a^	48 ± 0.05
pH 7.0	46 ± 0.15	50 ± 0.10

^a^ Data are presented as mean ± standard deviation. The number only considers the hydrogen bonds with more than 85% persistence during the simulation.

**Table 5:** Hydrogen bond pattern at the interface of the wild-type (WT-WT) and mutated (E326K-E326K) homodimer and of the heterodimer (WT-E326K) at pH 5.5.

Residue of Chain A	Residue of Chain B	WT-WT	E326K-E326K	WT-E326K
S12	S242	--	46.00 ^a^	--
Y244	F347	36.63	--	--
W348	Y244	37.84	--	--
E349	S242	--	--	60.02
E349	G243	--	--	44.07
E349	Y244	46.97	--	--
E350	G243	--	--	64.75
E350	S345	--	--	35.44
D358	S242	--	88.59	--
R395	E349	35.55	--	--
F397	F347	38.85	--	--
Number of total hydrogen bonds		5	2	4

^a^ The number indicates the percentage (%) of persistence of hydrogen bonds throughout the concatenated simulation. The value was calculated as the average of the persistence percentages of individual replicas that are reported in Table S4. Only the hydrogen bonds with a persistence of over 35% were considered. The residues involved in these hydrogen bonds are depicted in Figure S3.

**Table 6:** Hydrogen bond pattern at the dimer’s interface at pH 7.0.

Residue of Chain A	Residue of Chain B	WT-WT	E326K-E326K	WT-E326K
N192	D358	--	36.40 ^a^	--
S242	D358	44.20	43.20	43.94
Y244	Y313	--	--	42.20
Y244	F347	--	--	47.95
Y244	E349	42.25	--	--
E254	S464	--	44.75	39.82
E254	S465	--	--	37.25
E284	D315	--	--	39.18
Y291	D445	--	35.40	--
K293	D443	--	44.76	--
V294	D443	--	41.63	--
V295	D443	--	42.15	--
D315	L240	45.32	--	--
D315	G243	--	45.57	--
L317	A320	--	--	37.18
D358	S242	55.15	--	--
Number of total hydrogen bonds		4	8	7

^a^ The number indicates the percentage (%) of persistence of hydrogen bonds throughout the concatenated simulation. The value was calculated as the average of the persistence percentages of individual replicas that are reported in Table S5. Only the hydrogen bonds with a persistence of over 35% were considered. The residues involved in these hydrogen bonds are depicted in Figure S3.

**Table 7:** Predicted binding free energies of GCase dimers at acidic and neutral pH, calculated using the PRODIGY server. Data are presented as mean ± standard deviation.

Dimers	ΔG (kcal/mol) at pH 5.5	ΔG (kcal/mol) at pH 7.0
WT-WT	−18.89 ± 0.43	−15.70 ± 0.16
E326K-E326K	−14.55 ± 0.18	−14.72 ± 0.25
E326K-WT	−16.88 ± 0.22	−14.99 ± 0.24

**Table 8:** Intermolecular interactions (hydrogen bonds and salt bridges) between GCase monomers at pH 7.0 and Lysosomal integral membrane protein-2 (LIMP-2).

GCase Residue	LIMP-2 Residue	Wild-Type	E326K
L94	K153	--	10.96 ^a^
A95	K153	--	12.92
Q101	K153	--	27.79
Q101	Q 156	--	41.23
N102	Q156		80.61
N102	E146	32.97	--
K106	E146	23.19	--
**R163**	**E149**	**64.37 ^b^**	**--**
R163	A154	--	13.50
R163	Q156	--	17.80
Q166	E175	--	11.10
Number of total hydrogen bonds		3	8
Number of total salt bridges		1	0

^a^ The number indicates the percentage (%) of persistence of hydrogen bond throughout the concatenated simulation. The value was calculated as the average of the persistence percentages of individual replicas that are reported in Table S6. Only the hydrogen bonds with a persistence of over 10% were considered. ^b^ Bold text highlights residue pairs that form stable salt bridges.

**Table 9:** Intermolecular interactions (hydrogen bonds and salt bridges) between GCase monomers at pH 5.5 and Saposin C (SapC).

GCase Residue	SapC Residue	Wild-Type	E326K
G10	Y53	--	17.35 ^a^
R44	E48	12.95	--
R44	D51	--	20.14
S237	*E8 ^b^*	*19.86*	*--*
K321	*E13*	*--*	*11.12*
R329	C77	--	38.05
***K346***	***E24***	***--***	***53.65 ^c^***
F347	L76	16.67	--
W348	S55	--	13.31
*E 349*	*Y3*	*16.59*	*--*
R353	D51	--	48.53
*D358*	*K25*	*--*	*26.66*
*Q362*	*E24*	*--*	*24.62*
*D443*	*T15*	*--*	*10.90*
*D443*	*K16*		*20.31*
D443	S55	28.89	--
*D445*	*K16*	*--*	*12.32*
***D463***	***D19***	***--***	***88.08***
D463	S59	11.18	--
S465	E63		18.55
*K466*	*E8*	*--*	*15.00*
Number of total hydrogen bonds		6	14
Number of total salt bridges		0	2

^a^ The number indicates the percentage (%) of persistence of hydrogen bonds throughout the concatenated simulation. The value was calculated as the average of the persistence percentages of individual replicas that are reported in Table S7. Only the hydrogen bonds with a persistence of over 10% were considered. ^b^ Italic text highlights residues belonging to domain 1 (residues 6–34) of SapC. ^c^ Bold text highlights residue pairs that form stable salt bridges.

**Table 10:** Intermolecular interactions (hydrogen bonds and salt bridges) between GCase monomers at pH 5.5 and α-Synuclein (α-Syn).

GCase Residue	α-Syn Residue	Wild-Type	E326K
Y11	P133	16.40 ^a^	--
Y11	E138	17.27	--
**R48**	**E130**	**31.80 ^b^**	**--**
Y313	E131	--	21.32
S345	K127	--	12.06
**K346**	**E131**	**31.07**	**--**
W348	L121	21.50	--
S351	E138	18.81	--
R353	E138	22.11	--
Number of total hydrogen bonds		7	2
Number of total salt bridges		2	0

^a^ The number indicates the percentage (%) of persistence of hydrogen bonds throughout the concatenated simulation. The value was calculated as the average of the persistence percentages of individual replicas that are reported in Table S8. Only the hydrogen bonds with a persistence of over 10% were considered. ^b^ Bold text highlights residue pairs that form stable salt bridges.

**Table 11:** Predicted binding free energies of the GCase monomer at neutral pH in complex with LIMP-2 and at acidic pH in complex with SapC and α-Syn, calculated using the PRODIGY server. Data are presented as mean ± standard deviation.

	ΔG (kcal/mol) Wild-Type	ΔG (kcal/mol) E326K
LIMP-2	−6.73 ± 0.14	−7.07 ± 0.12
SapC	−7.60 ± 0.21	−7.86 ± 0.14
α-Syn	−9.31 ± 0.21	−7.47 ± 0.50

**Table 12:** Summary of simulations performed.

Simulation Name	Description of the System	Simulation Length
WT-WT_pH5	Wild-type GCase homodimer at pH 5.5	1000 ns, 2 replicas ^a^
E326K-E326K_pH5	Mutated GCase homodimer at pH 5.5	1000 ns, 2 replicas
WT-E326K_pH5	GCase heterodimer at pH 5.5	1000 ns, 2 replicas
WT-WT_pH7	Wild-type GCase homodimer at pH 7.0	1000 ns, 2 replicas
E326K-E326K_pH7	Mutated GCase homodimer at pH 7.0	1000 ns, 2 replicas
WT-E326K_pH7	GCase heterodimer at pH 7.0	1000 ns, 2 replicas
WT-LIMP2	Wild-type GCase monomer at pH 7.0 in complex with LIMP-2 (endoplasmic reticulum interaction)	200 ns, 3 replicas ^b^
E326K-LIMP2	Mutated GCase monomer at pH 7.0 in complex with LIMP-2 (endoplasmic reticulum interaction)	200 ns, 3 replicas
WT-SapC	Wild-type GCase monomer at pH 5.5 in complex with Saposin C (lysosomal interaction)	200 ns, 3 replicas
E326K-SapC	Mutated GCase monomer at pH 5.5 in complex with Saposin C (lysosomal interaction)	200 ns, 3 replicas
WT-α-Syn	Wild-type GCase monomer at pH 5.5 in complex with α−Synuclein (lysosomal interaction)	200 ns, 3 replicas
E326K-α-Syn	Muated GCase monomer at pH 5.5 in complex with α-Synuclein (lysosomal interaction)	200 ns, 3 replicas

^a^ Replicas’ frames were concatenated to form a single continuous trajectory for each system after discharging the first 400 ns of simulation (equilibration phase). ^b^ Replicas’ frames were concatenated to form a single continuous trajectory for each system after discharging the first 50 ns of simulation (equilibration phase).

## Data Availability

The dataset is available on request from the authors.

## Supplementary Materials

The following supporting information can be downloaded at: https://www.mdpi.com/1422-0067/25/21/11443/s1.
