# Supplementary material for: Investigating the Impact of the Parkinson’s-Associated GBA1 E326K Mutation on β-Glucocerebrosidase Dimerization and Interactome Dynamics Through an In Silico Approach
Source: Int J Mol Sci. 2024 Oct 24;25(21):11443. doi: 10.3390/ijms252111443 (PMC11870034; doi:10.3390/ijms252111443)
Supplement: Supplementary file 1 [file ijms-25-11443-s001.zip › ijms-3233068-supplementary.pdf]

Supplementary Materials

Investigating the impact of the Parkinson's-associated GBA1 E326K mutation on GCase dimerization and interactome dynamics through an *in silico* approach

Davide Pietrafesa [1,2], Alessia Casamassa [3], Barbara Benassi [4], Massimo Santoro [4], Massimo Marano [5,6], Claudia Consales [4], Jessica Rosati [3,7] and Caterina Arcangeli [4,*]

[1] Department of Biology, University of Rome Tor Vergata, Via della Ricerca Scientifica 1, 00133 Rome, Italy
[2] PhD Program in Materials for Health, Environment and Energy, University of Rome Tor Vergata, Via della Ricerca Scientifica 1, 00133 Rome, Italy; email
[3] Cellular Reprogramming Unit, Fondazione IRCCS Casa Sollievo della Sofferenza, Viale dei Cappuccini 1, 71013 San Giovanni Rotondo, Foggia, Italy
[4] Division of Biotechnologies, Department for Sustainability, Italian National Agency for New Technologies, Energy and Sustainable Economic Development (ENEA), Via Anguillarese 301, 00123 Rome, Italy
[5] Unit of Neurology, Neurophysiology, Neurobiology and Psychiatry, Department of Medicine, Università Campus Biomedico, Via Alvaro del Portillo 200, 00128, Rome, Italy
[6] Fondazione Policlinico Universitario Campus Biomedico, Via Alvaro del Portillo 200, 00128, Rome, Italy
[7] Departmental Faculty of Medicine, Saint Camillus International University of Health Sciences, Via di Sant'Alessandro 8,00131 Rome, Italy
* Correspondence: caterina.arcangeli@enea.it; Tel.: +39 (06)3048.6898

Table S1. Time averaged distances (nm) between specific residues at pH 7.0.

Notes: Data are presented as mean ± standard deviation

Simulation at pH 7.0	WT	E326K
**E/K326 – E235**	2.33 +/- 0.07	2.10 +/- 0.08
**E/K326 – E340**	2.29 +/- 0.07	1.89 +/- 0.09
**E235 – E340**	0.41 +/- 0.04	0.52 +/- 0.07

Table S2. Hydrogen bonds between specific residues of the active site.

Notes: The number indicates the persistence (%) of hydrogen bond throughout the single replica.

Pair residues, pH	WT		E326K	
	Replica 1	*Replica 2*	*Replica 1*	*Replica 2*
**Y313-E235, pH 7.0**	90.84	44.56	--	--
**Y313-E340, pH 5.5**	--	--	83.58	12.02

Table S3. Hydrogen bond involving the mutation site, residue 326 at neutral pH.

Notes: The number indicates the persistence (%) of hydrogen bond throughout the single replica.

**pH 7.0**	**WT**		**E326K**	
	Replica 1	***Replica 2***	***Replica 1***	***Replica 2***
**Residue/Residue**	*E326*		*K326*	
H328	57.46	70.62	--	--
R329	85.35	79.49	--	--
L330	78.64	74.00	80.84	80.72

Table S4. Hydrogen bond pattern at the dimer's interface at pH 5.5.

Notes: The number indicates the persistence (%) of hydrogen bond throughout the single replica.

**Residue of Chain A**	**Residue of Chain B**	**WT-WT**		**E326K-E326K**		**WT-E326K**	
		Replica 1	***Replica 2***	***Replica 1***	***Replica 2***	***Replica 1***	***Replica 2***
S12	S242	--	--	0.10	90.77	--	--
Y244	F347	62.21	11.04	--	--	--	--
W348	Y244	69.07	6.60	--	--	--	--
E349	S242	--	--	--	--	81.30	38.74
E349	G243	--	--	--	--	86.18	0.01
E349	Y244	93.93	0.00	--	--	--	--
E350	G243	--	--	--	--	79.32	50.19
E350	S345	--	--	--	--	64.33	62.81
D358	S242	--	--	84.00	90.87	--	--
R395	E349	20.03	51.07	--	--	--	--
F397	F347	6.12	71.57	--	--	--	--

Table S5. Hydrogen bond pattern at the dimer's interface at pH 7.0.

Notes: The number indicates the persistence (%) of hydrogen bond throughout the single replica.

**Residue of Chain A**	**Residue of Chain B**	**WT-WT**		**E326K-E326K**		**WT-E326K**	
		Replica 1	***Replica 2***	***Replica 1***	***Replica 2***	***Replica 1***	***Replica 2***
N192	D358	--	--	0.00	72.75	--	--
S242	D358	80.67	7.73	70.27	16.13	84.52	3,36
Y244	Y313	--	--	--	--	14.08	80.32
Y244	F347	--	--	--	--	4.05	80.10
Y244	E349	11.02	73.48	--	--	--	--
E254	S464	--	--	81.38	8.12	0.00	70.89
E254	S465	--	--	--	--	34.02	40.48
E284	D315	--	--	--	--	78.35	0.00
Y291	D445	--	--	70.79	0.00	--	--
K293	D443	--	--	80.50	9.02	--	--
V294	D443	--	--	83.24	0.00	--	--
V295	D443	--	--	81.16	3.12	--	--
D315	L240	86.23	4.40	--	--	--	--
D315	G243	--	--	0.00	91.23	--	--
L317	A320	--	--	--	--	5.12	69.23
D358	S242	39.85	70.45	--	--	--	--

Table S6. Hydrogen bonds between GCase monomers at pH 7.0 and LIMP-2.

Notes: The number indicates the persistence (%) of hydrogen bond throughout the single replica.

GCase residue	*LIMP-2 residue*	*Wild-type*			*E326K*		
		Replica 1	***Replica 2***	***Replica 3***	***Replica 1***	***Replica2***	***Replica 3***
L94	K153	--	--	--	15.33	10.30	7.25
A95	K153	--	--	--	9.90	28.86	0.00
Q101	K153	--	--	--	26.93	42.72	13.72
Q101	Q 156	--	--	--	40.09	38.76	44.84
N102	Q 156	-	-	-	83.82	89.12	68.89
N102	E146	19.51	41.99	37.41	-	-	-
K106	E146	55.48	6.54	7.55	--	--	--
R163	E149	79.64	78.71	34.76	--	--	--
R163	A154	--	--	--	0.00	26.36	14.14
R163	Q156	--	--	--	0.00	12.90	40.50
Q166	E175	--	--	--	0.00	25.66	7.64

Table S7. Hydrogen bonds between GCase monomers at pH 5.5 and Sap-C.

Notes: The number indicates the persistence (%) of hydrogen bond throughout the single replica.

GCase residue	*Sap-C residue*	*Wild-type*			*E326K*		
		Replica 1	*Replica 2*	*Replica 3*	*Replica 1*	*Replica2*	*Replica 3*
G10	Y53	--	--	--	42.01	10.04	0.00
R44	E48	26.32	2.50	10.03	--	--	--
R44	D51	--	--	--	0.00	0.00	60.42
S237	E8	10.12	49.55	0.00	--	--	--
K321	E13	--	--	--	0.00	0.02	33.38
R329	C77	--	--	--	12.40	28.40	73.35
K346	E24	--	--	--	88.40	72.51	0.04
F347	L76	0.03	35.96	14.02	--	--	--
W348	S55	--	--	--	25.09	10.62	4.22
E 349	Y3	10.02	39.75	0.00	--	--	--
R353	D51	--	--	--	71.65	62.41	11.53
D358	K25	--	--	--	11.02	0.00	68.96
Q362	E24	--	--	--	0.00	9.54	64.29
D443	T15	--	--	--	0.08	32.62	0.00
D443	K16				18.23	31.76	10.94
D443	S55	0.00	17.03	69.70	--	--	--
D445	K16	--	--	--	0.38	0.00	36.58
D463	D19	--	--	--	74.11	90.90	99.23
D463	S59	0.00	9.01	24.53	--	--	--
S465	E63				0.00	55.61	0.04
K466	E8	--	--	--	44.99	0.01	0.00

Table S8. Hydrogen bonds between GCase monomers at pH 5.5 and α-syn.

Notes: The number indicates the persistence (%) of hydrogen bond throughout the single replica.

GCase residue	*a-syn residue*	***Wild-type***			***E326K***		
		Replica 1	***Replica 2***	***Replica 3***	***Replica 1***	***Replica2***	***Replica 3***
Y11	P133	22.15	14.80	12.25	--	--	--
Y11	E138	10.11	7.39	34.31	--	--	--
R48	E130	23.12	35.56	36.72	--	--	--
Y313	E131	--	--	--	0.02	11.52	52.42
S345	K127	--	--	--	0.01	17.53	18.64
K346	E131	22.63	30.33	40.25	--	--	--
W348	L121	11.03	33.13	20.34	--	--	--
S351	E138	5.90	42.55	7.98	--	--	--
R353	E138	8.82	9.59	47.92	--	--	--

Table S9. List of the titratable residues in GCase.

Notes: Predicted protonation states of titratable residues in GCase at pH 7.0 and 5.5. HISA, H at δ-nitrogen (neutral); HISB, H at ε-nitrogen (neutral); HISH, H at both nitrogens (charged); ASPH, protonated aspartic acid; GLUH, protonated glutamic acid.

Residue (number)	Residue (name)	pH 7.0	pH 5.5
60	HIS	HISA	HISH
87	ASP	ASP	ASPH
145	HIS	HISA	HISH
162	HIS	HISA	HISH
206	HIS	HISB	HISA
223	HIS	HISA	HISH
274	HIS	HISA	HISH
283	ASP	ASP	ASPH
290	HIS	HISA	HISH
328	HIS	HISA	HISH
340	GLU	GLU	GLUH
365	HIS	HISA	HISH
451	HIS	HISB	HISA
490	HIS	HISA	HISB
495	HIS	HISA	HISH

Tabella S10. Two-step equilibration protocol.

Notes: a = simulation time (ps); b = timestep (fs); c = temperature (K); d = force constant for position constraints (kJ/mol-1/nm-2); e = time constant for thermal coupling of solute (a) and solvent (b) (ps); f = time constant for coupling to barostat (ps)

	**Time**[a]	**Δt**[b]	**T**[c]	**F_c (x 10^3)**[d]	**T_t**[e,]		**T_t**[f]
**NVT**	200	2	100	1000	0.1[(a)]	0.1[(b)]	-
**NPT**	200	2	310	1000	0.1	0.1	2.0

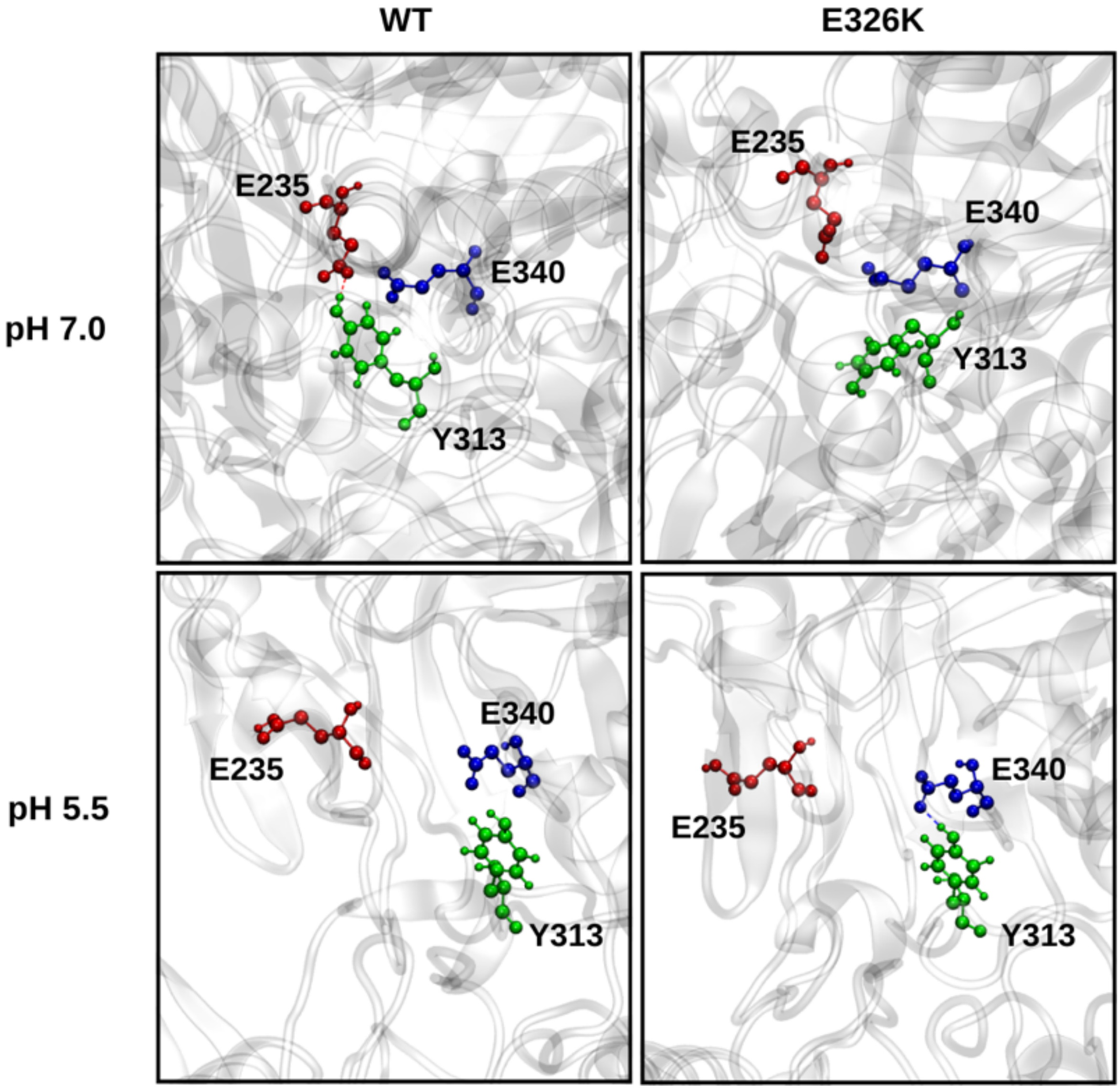

Figure S1. Hydrogen bonds between specific residues of the active site. The hydrogen bonds between the catalytic residues (E235 and E340) and the Y313 residue of the wild-type and mutated GCase at pH 7.0 and 5.5 reported in Table 2 are here depicted as dashed lines.

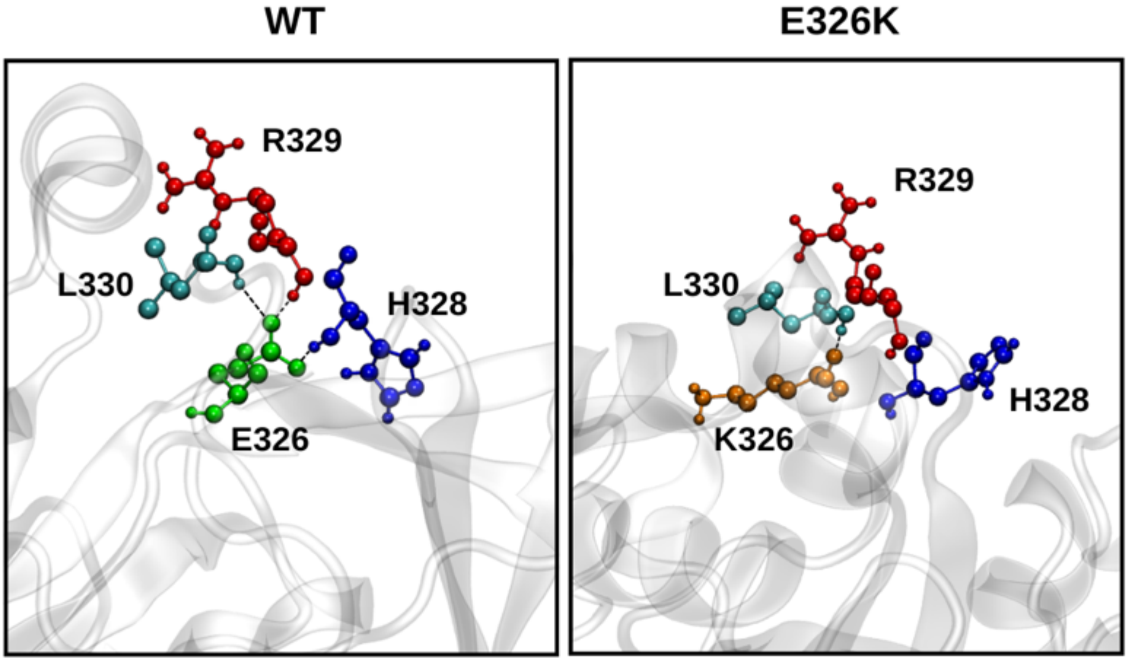

Figure S2. Hydrogen bonds involving the mutation site, residue 326, at neutral pH. The hydrogen bonds reported in Table 3 are here depicted as dashed lines.

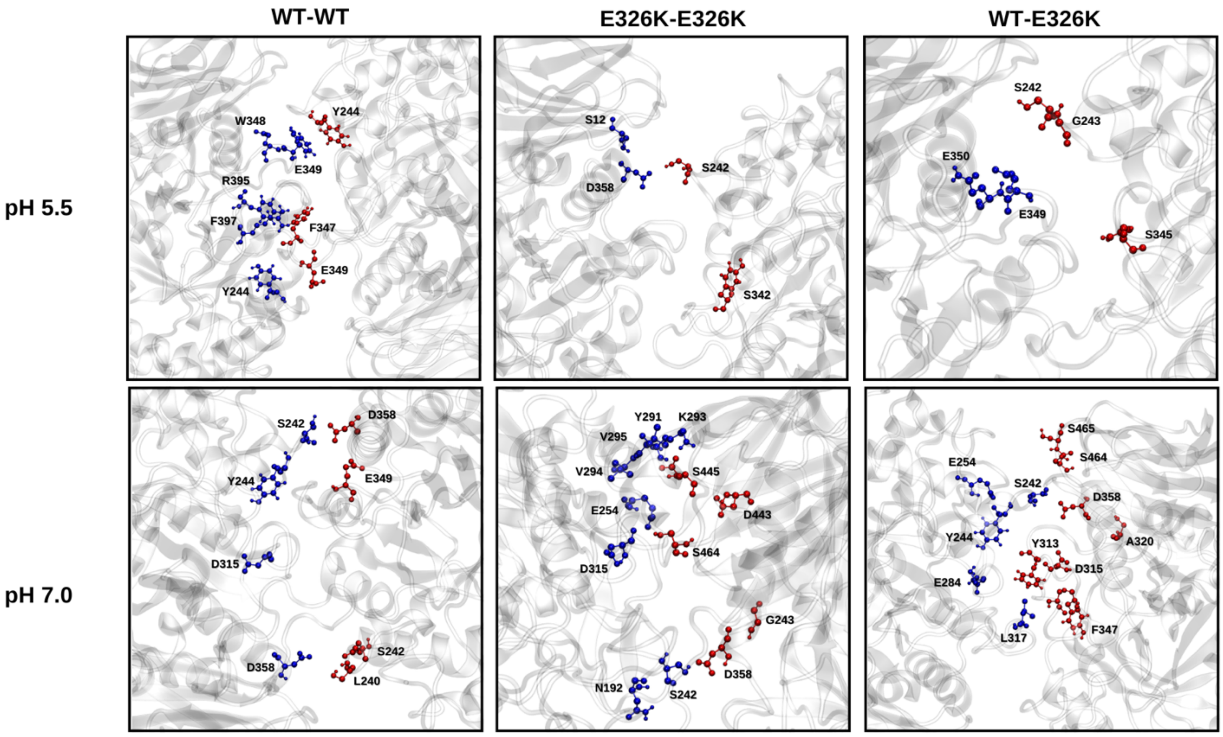

Figure S3. Residues involved in hydrogen bond patterns at the interface of the GCase dimers at acidic and neutral pH. The residues involved in interchain hydrogen bonds indicated in Table 5 and Table 6 are here shown and labelled: residues of chain A and chain B are colored in blue and red, respectively.

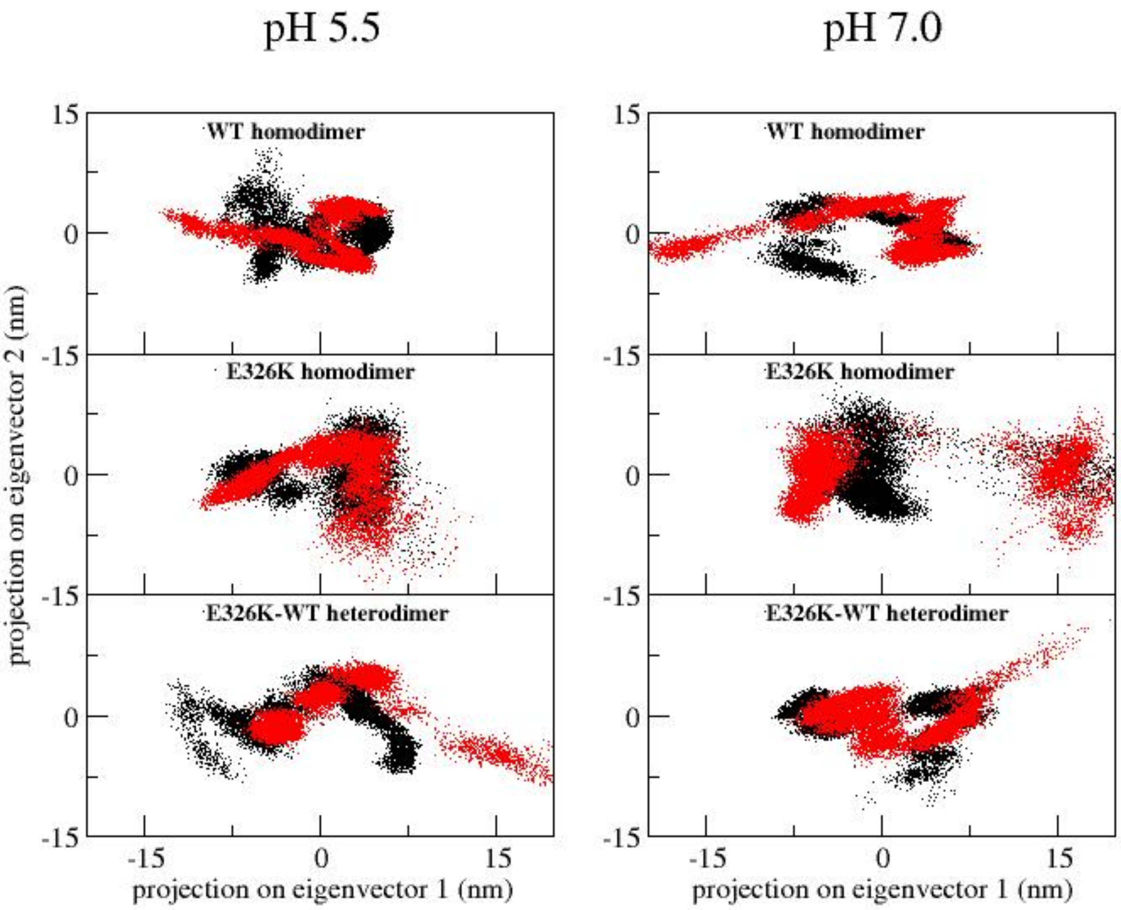

Figure S4. Projections of the dimers MD trajectories onto 2D space spanned by the first (PC1) and second principal axes (PC2). MD simulation replica 1 is represented in black, MD replica 2 is represented in red.

The two simulation replicas of each dimer explore similar conformational spaces, indicating reproducibility of the system's dynamics. At pH 5.5 the WT homodimer mainly occupies a single cluster, with minimal variability near the origin, indicating a stable conformational state, whereas at pH 7.0 it explores multiple conformational states, as shown by the broader distribution along both PC1 and PC2. The E326K homodimer shows greater spread along PC1 at pH 5.5 compared to the WT, indicating enhanced flexibility but still without distinct cluster separation, remaining in a single, more dynamic state. At pH 7.0, the E326K homodimer occupies two main conformational states (two clusters along PC1), suggesting that the mutation promotes transitions between at least two predominant conformations, leading to greater conformational heterogeneity. The E326K-WT heterodimer spans a broad area without distinct clusters at pH 5.5, suggesting it occupies a single, flexible conformational state with dynamic fluctuations. At pH 7.0, it exhibits a more concentrated distribution with two defined clusters, indicating that it explores two primary conformational states, showing some stabilization and potentially reduced flexibility compared to pH 5.5.

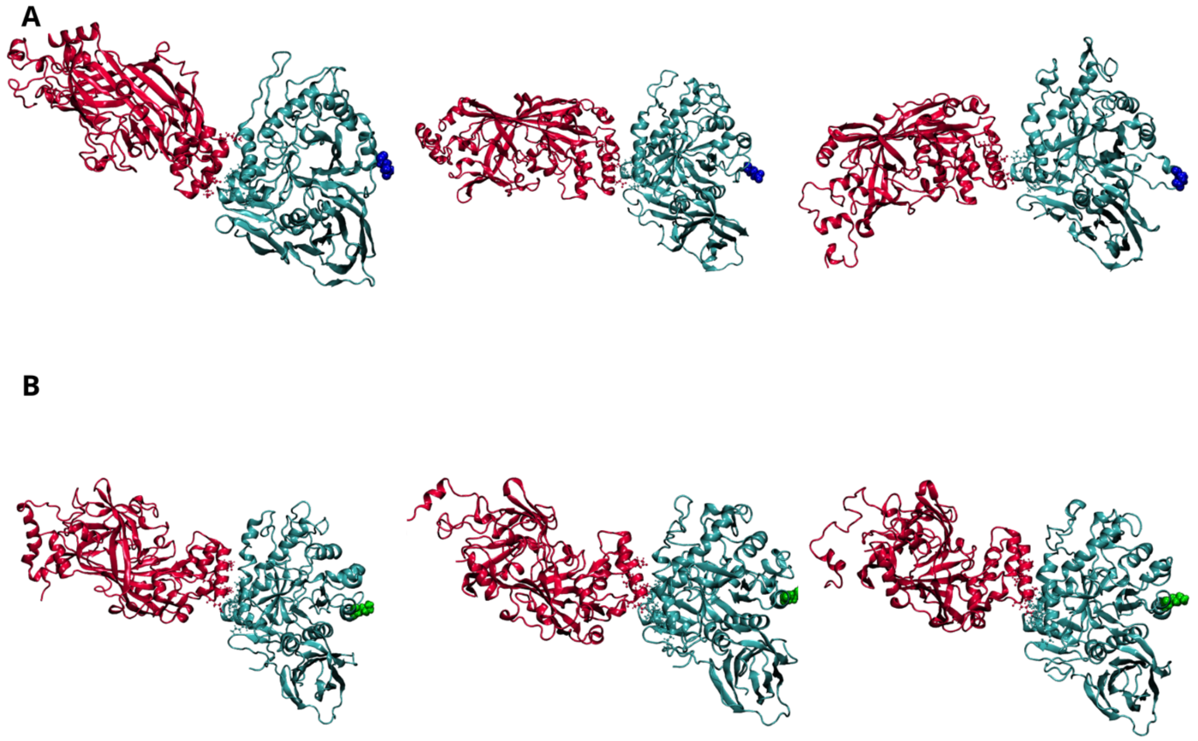

Figure S5. Dynamics of interaction between GCase and LIMP-2 at pH 7.0. Representative snapshots from the concatenated MD trajectories of the WT-LIMP-2 complex (A) and the E326K-LIMP-2 complex (B). GCase is shown in cyan, and LIMP-2 is in red, both depicted in a New Cartoon representation. Key residues involved in the interaction between the two proteins are displayed in the CPK model. The mutation site, residue 326, is highlighted in blue for the wild-type GCase and green for the mutated GCase, represented in the VDW model. Here the entire structure of LIMP-2 is shown; however for clarity the water molecules are not shown.

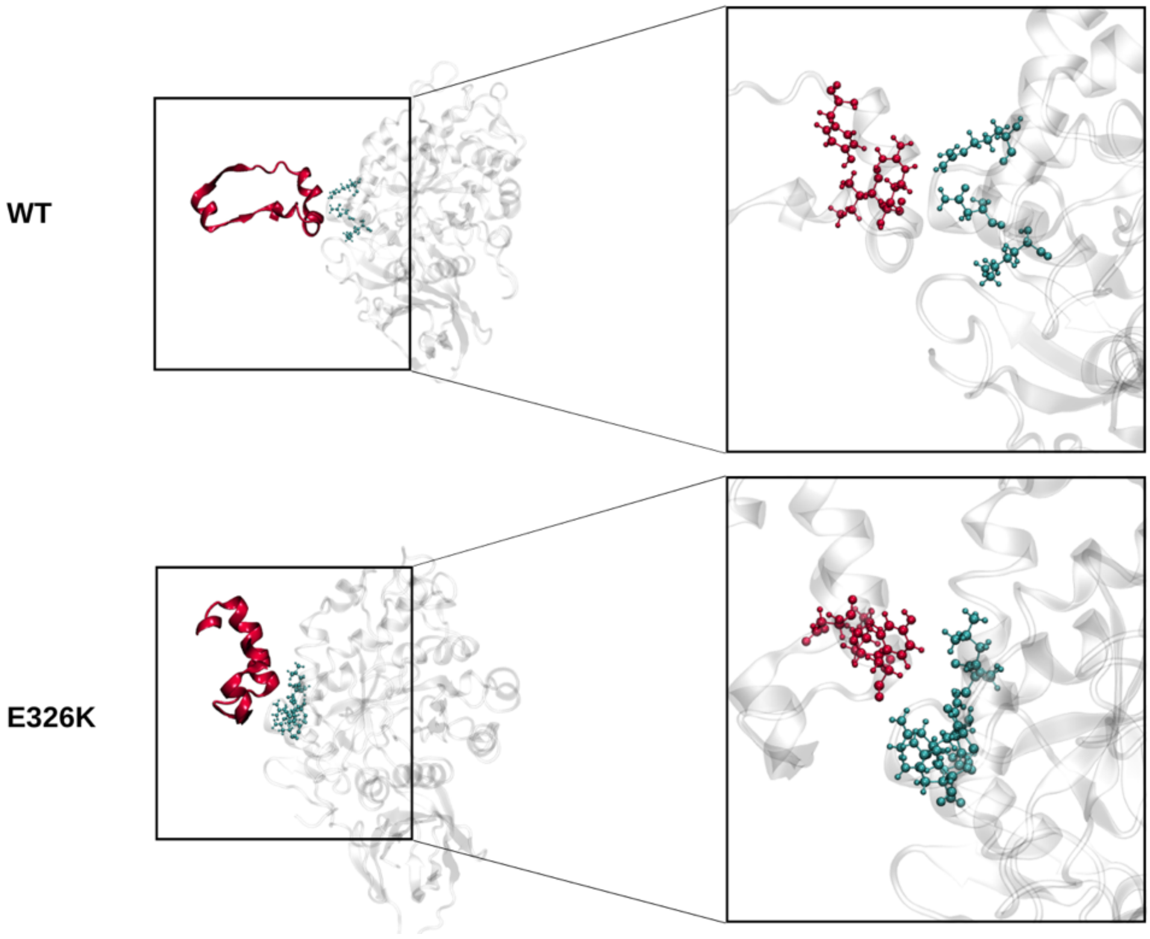

Figure S6. Residues involved in GCase-LIMP-2 interaction at pH 7.0. Representative snapshot from the concatenated MD trajectories of the WT-LIMP-2 complex (upper) and the E326K-LIMP-2 complex (lower). GCase is shown in cyan, and LIMP-2 is in red. Zoom area: key residues involved in the interaction between the two proteins are displayed in the CPK model.

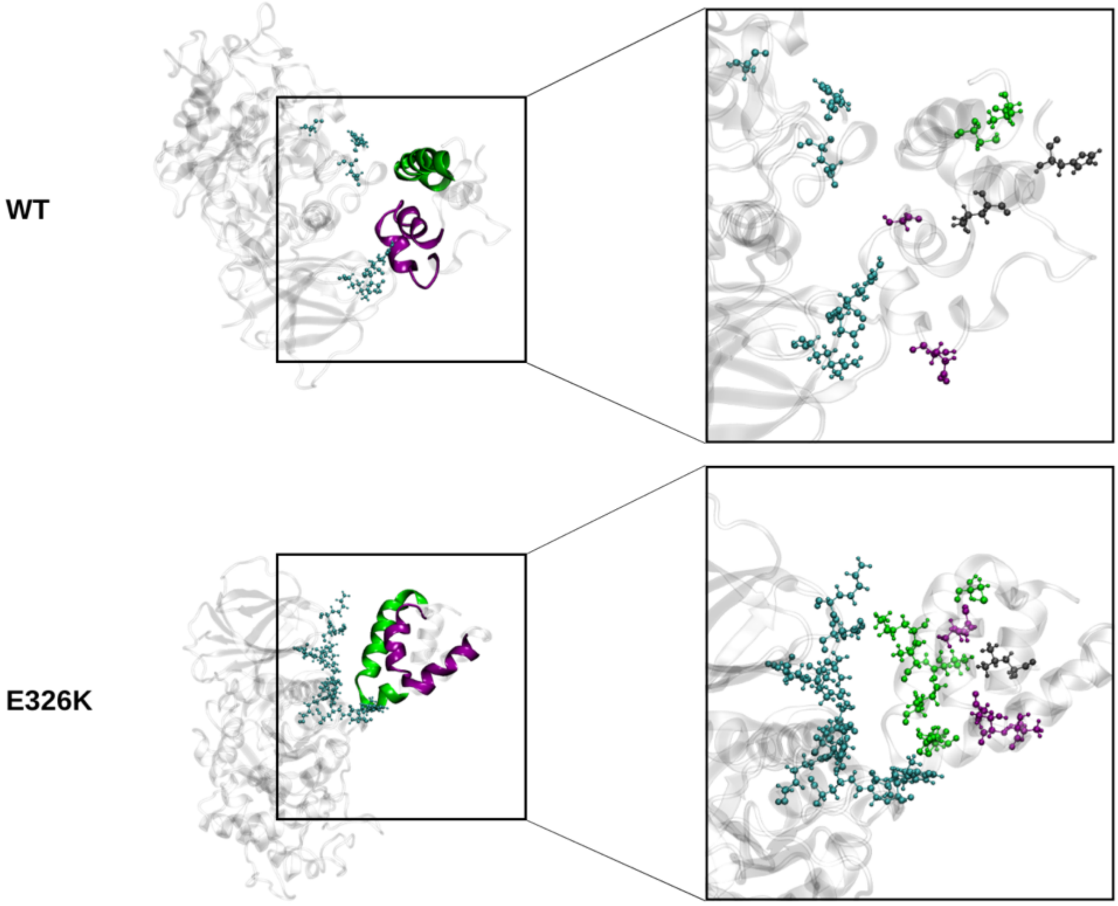

Figure S7. Residues involved in GCase-SapC interaction at pH 5.5. Representative snapshot from the concatenated MD trajectories of the WT-SapC complex (upper) and the E326K-SapC complex (lower). GCase is shown in cyan, and domain I and II of SapC are in green and purple, respectively. Zoom area: key residues involved in the interaction between the two proteins are displayed in the CPK model.

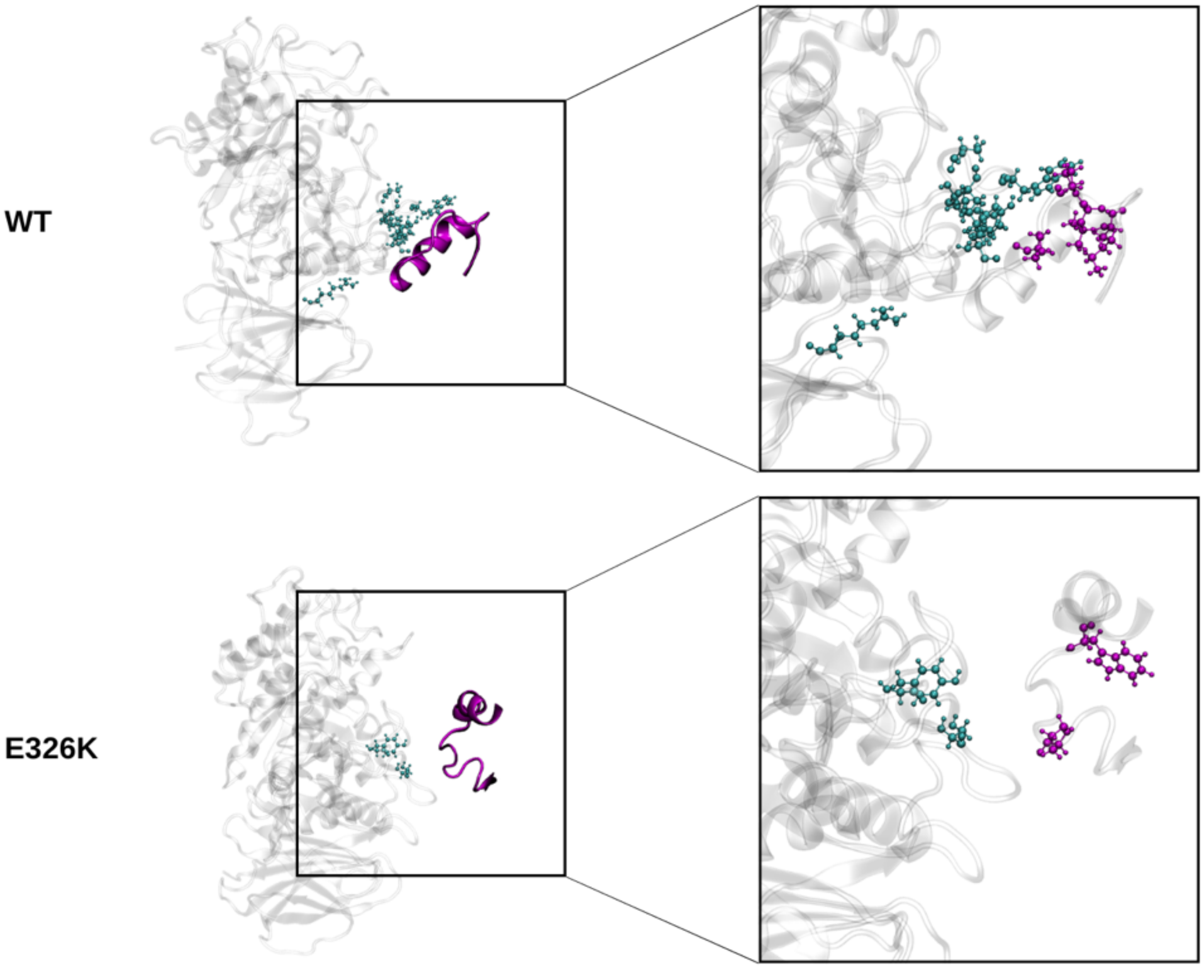

Figure S8. Residues involved in GCase-α-syn interaction at pH 5.5. Representative snapshot from the concatenated MD trajectories of the WT-α-syn complex (upper) and the E326K-α-syn complex (lower). GCase is shown in cyan, and α-syn is in purple. Zoom area: key residues involved in the interaction between the two proteins are displayed in the CPK model.
